# The Organization in Micro-Loops of an Extended Fragment of Chicken Chromosome 14, Including the Alpha Globin Gene Cluster in the Erythroid Cells

**Published:** 2009-04

**Authors:** E.S. Philonenko, A.A. Gavrilov, S.V. Ravin, O.V. Iarovaia

**Affiliations:** 1 Institute of Gene Biology, Russian Academy of Sciences, 34/5 Vavilov Street, 119344 Moscow, Russia

## Abstract

It has been shown that the activation of tissue-specific gene transcription during the course of cell differentiation is associated with a spatial reorganization of the genomic domains harboring those specific genes. This reorganization consists of the relocation to the nuclear matrix of the whole genomic domain containing one or more of the genes being transcribed. However, it remains unclear whether, during this process, extended areas of the genome also become attached to the nuclear matrix. We studied the genome´s pattern of interaction with the nuclear matrix in both erythroid and non-erythroid cells of chickens, using a 220Kb region of chromosome #14, which contains the alpha-globin gene cluster and some surrounding house-keeping genes. The results show that in erythroid cells, the fragment of the genome containing the alpha-globin gene domain became spatially arranged into micro-loops which could not be detected by mapping experiments.

In eukaryotic cells, chromosomal DNA is organized into a series of loops which are attached to the nuclear matrix [1, 2]. The attachment sites for DNA on the nuclear matrix are all different, yet it is possible to distinguish between permanent (stable, structural) DNA attachment sites, which exist in cells of different lineages, and facultative (i.e. temporary, functional, tissue-specific) attachment sites, which are found only in cells in a particular lineage or during a particular stage of differentiation [2, 5]. In the present study, we characterized the spatial organization of a large (220 Kb) segment of the chicken chromosome 14, which includes a cluster of erythroid-cell specific alpha-globin genes, as well as a number of open reading frames (Fig. 1) [6]. For the purpose of distinguishing between the various types of interactions between DNA and the nuclear matrix, the spatial organization of the above-mentioned region was studied in both erythroid- and non-erythroid cells. Virus-transformed chicken erythroblasts, from the HD3 cell line (A6 clone of the LSCC cell line), and cells from the lymphoid cell line DT40 (CRL-2111, ATCC) were used as cellular models. 

**Fig. 1.**
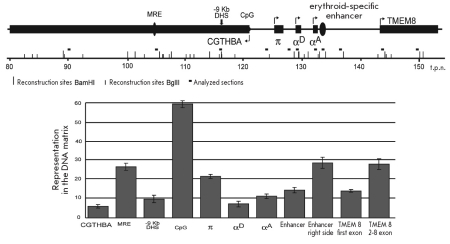
Mapping attachment sites of DNA to the nuclear matrix for a 220 Kb segment of chicken chromosome 14, which includes the α-globin gene cluster. At the top of the figure, a scheme of the genome area under study is shown. Within the scheme: RHBDF - a gene encoding the epidermal growth factor receptor; MPG - a gene encoding the N-methylpurine-DNA glycosylase; СGTHBA - a housekeeping gene of unknown function; СpG - CpG island containing an origin of DNA replication; π - embryonic α-type globin gene; αA and αD "adult" α - globin genes; TMEM8 - an ORF encoding an unknown transmembrane protein; P15 - a gene encoding mitochondrial ribosomal protein L28; Axin1 - a gene encoding axin 1. Kb - thousand pairs of nucleotides. In the bottom part of the figure, the results of mapping sites of DNA attachment to the nuclear matrix are presented in the form of a graph (see the text for details). The black rectangle represents the extended area, which appears entirely attached to the nuclear matrix in HD3 cells.

In previous experiments with lymphoid cells, using in-situ hybridization with nuclear halos of BAC-probes representing the analyzed area of the genome, we have shown that this area is spatially arranged in small loops [8]. However, in erythroid cells, the spatial arrangement of the same area of the genome is quite different; here the entire genomic segment being studied collapsed onto the nuclear matrix and could be visualized as a dot following in-situ hybridization of the corresponding DNA BAC clone with the nuclear halos [8]. 

It has been previously reported that active, tissue-specific genes associate with the nuclear matrix [9-11]. In this respect it seemed only logical to assume that the collapse of the DNA loop which was observed in chicken erythroblasts was a consequence of the activation of tissue-specific genes during the course of erythroid differentiation. In order to obtain a map of the DNA interaction with the nuclear matrix for the area of the genome currently being studied, we adopted the following approach: short test fragments were distributed over the whole length of the genome being studied, with a distance of 5 Kb separating consecutive test fragments, and then the relative representation of each of these test fragments in the nuclear matrix DNA (compared to the total DNA) was determined using a semi-quantitative PCR analysis (Fig. 1). The primer sequences used for these PCR reactions are listed in Table 1. 

**Table1:** List of the primers used in semi-quantitative PCR for mapping sites of DNA attachment to the nuclear matrix

name	Sequence
01 dir	5' CTCATTTGCCAGCGAGATAT 3'
01 rev	5' GCCTCGATGGTGCAGTAAGC 3'
02 dir	5' КCCCTTGTAGGCTGCAACCCG К3'
02 rev	5' КACAGTCCCTTTTCCATCACC К3'
03 dir	5' КCATCTGTGCATCCGTTCTAC К3'
03 rev	5' КTCTTCTAAAGTGCCACCATC К3'
04 dir	5' КCCCTATTTTCAGGGTTATTA К3' К К
04 rev	5' КAAATGTAAAGCGATTGGTAG К3'
05 dir	5' КGCGTGGAGTAGTTCAGGTAA К3'
05 rev	5' КGACACTGAGTCCCACCAAAG К3'
06 dir	5' КCACTTGTGAATGCAGGTTAA К3'
06 rev	5' КTCTTCTAAGATTGCCGTGTT К3'
07 dir	5' КGGCTGCATAGCATTACTTTC К3'
07 rev	5' КGTAGGACTTAACACCAACGT К3'
08 dir	5' КTCCATGTAAGGAGCAGATTT К3'
08 rev	5' КGTGTTTTGGGAGCAGTGAGT К3'
09 dir	5' КTCATTCTGCCTGACCACTTT К3'
09 rev	5' КCCTCCTCAGAGCCAACCAAA К3'
10 dir	5' КGGAAACTCAGGGCTCACAAT К3'
10 rev	5' КGCAAGGCCAAGAAGCAATAT К3'
11 dir	5' КAAGCACTCAGAAGCACAACA К3'
11 rev	5' КGCCAGACTTACGAAATCAAA К3'
12 dir	5' КCACAGGACTATCCAGGTATG К3'
12 rev	5' КAGGGCTGTCAGTCTTCAGTA К3'
13 dir	5' GCTGCTCTACCTTGTTCTCA 3'
13 rev	5' GCCTTGTTCTCTTCCCTACC 3'
14 dir	5' CTGCCTCATGTTTGTTAAGA 3'
14 rev	5' CAAAGTCCCAAAAGCTATAA 3'
15 dir	5' ATTACCAAGCCTACTTCATT 3'
15 rev	5' GTTGAGACTTTGATCTGTGG 3'
16 dir	5' CAGAGCTCAATTCCATAGG 3'
16 rev	5' TTATCTGGGGTACCTGCAT 3'
17 dir	5' КTGTTCCCTGGTACTCGTCAG К3'
17 rev	5' КTCACCGCATATCGACTCCGT К3'
18 dir	5' GCAGACTCTTAGATTGGCAT 3'
18 rev	5' CTCAGTCAGAACAGAGGAAA 3'
19 dir	5' GTGAAAAAAATCCACTGTAAA 3'
19 rev	5' ATCTAAAGCCAATGAAGAAAA 3'
20 dir	5' TATCCCTCCCTGCCTTACCC 3'
20 rev	5' AGGCAGCCACTACCTTCTTG 3'
21 dir	5' GCCCTTCGTGTCCTTGATTT 3'
21 rev	5' ATTCCAGCAGCCTTTCTTCC 3'
22 dir	5' ACCTCATCACCCTTCCACAT 3'
22 rev	5' TGCCACAAACCATCGTCTCA 3'
23 dir	5' TCAGGAGCACCACCTTTAGA 3'
23 rev	5' TTGTGGCAGCACTTCGGTAA3'
24 dir	5' GGAGTGCTACTTCCTTTGAT 3'
24 rev	5' TGGTAATGTTCCTACTGGGT 3'
25 dir	5' AAGCGTGGTGCATGTGGAAA 3'
25 rev	5' TGAGTCTGCTGATGGGTCTG 3'
26 dir	5' КTCCCTAACAATGTGAGTTCC К3'
26 rev	5' КCTGCTGCATACAGTCTTGGA К3'
27 dir	5' КGTGCTTATCTGAGCCCTTCC К3'
27 rev	5' КCAGTATCACCCAGCTCCACA К3'
28 dir	5' КTTCAAATCACTTACGCTACA К3'
28 rev	5' КCTGGTTATCTGCCTACTCTG К3'
29 dir	5' КTGGATGCTGACAGTGCTTGA К3'
29 rev	5' КTGCGGTGAAAGAGTTGGAGT К3'
30 dir	5' КCAGTCTATCTCCCGTTGCTA К3'
30 rev	5' КAAACCTTACGGCTGGTCTCA К3'

The nuclear matrix DNA was recovered as described [11-12], employing a relatively mild nuclease treatment, so that the average size of the nuclear matrix DNA fragments recovered was ~ 5 Kb. The results of this analysis are presented in Fig. 1, as a ratio of the concentration of each test fragment in the total DNA versus its concentration in the nuclear matrix DNA. On the graph shown in Fig. 1, the regions bound to the nuclear matrix would be expected to lie close to the x-axis, while regions arranged in loops should be represented by peaks. The regions containing the CpG island upstream from the α-globin gene domain were previously shown to be permanently bound to the nuclear matrix [5] and thus served as a kind of internal positive control. An analysis of the results shown in Fig. 1 suggests that in the DT40 lymphoid cell line the attachment sites on the matrix DNA are distributed regularly along the entire length of the genome being studied, at an average distance of approximately 20 to 30 Kb. These results are in keeping with previous data obtained using a different experimental approach [8]. In erythroid cells, the larger part of the genome area under study (fragment corresponding to the coordinates 95-165 Kb in Fig. 1) appeared attached to the nuclear matrix. Within this fragment, there was no alternation of nuclear matrix-bound regions and unbound regions, while in the segments flanking this region, this alternating arrangement can be clearly seen. The long DNA fragment attached to the nuclear matrix harbors the cluster of α-globin genes, as well as genes P15, TMEM8, and a part of gene CGTHBA. The spatial arrangement of this segment of the genome, whose coordinates in Fig. 1 are 35 - 95 Kb, is similar in both HD3 and DT40 cell lines. This area of the genome also contains housekeeping genes that are transcribed at a low rate in both erythroid and lymphoid cells [13]. All of the results obtained correlate well with previous data showing the spatial arrangement of the alpha-globin gene cluster and the flanking areas of the genome obtained using galo-FISH [8]. It remains unclear, however, whether the area of the genome represented by coordinates 95 - 165 Kb (Fig. 1) is entirely attached to the nuclear matrix. An alternative model suggests that this area of the genome is spatially arranged into small loops (micro-loops) which cannot be recognized in our experiments due to the relatively large (5 Kb) distances between the test fragments, and the large size of the matrix DNA fragments. In order to test this possibility, we conducted a more detailed analysis of the association of several functionally important regions within the area under study and the nuclear matrix. In these experiments, the HD3 erythroid cells were used as the cellular model. The nuclear matrix DNA was obtained after the distal portions of the DNA loops were cleaved using the restriction enzymes Bam HI and Bam HII. The relative representation of DNA fragments containing globin genes and different regulatory elements within this preparation of matrix DNA was determined by real-time PCR analysis.

The nucleotide sequences of the TaqMan probes and the PCR primers used are presented in Table II. The results (diagram in Fig. 2) demonstrate that the relative representation in the nuclear matrix DNA of different DNA fragments from the apparently attached area was not the same. DNA fragments containing the CpG island upstream from the α-globin gene domain, the central part of the TMEM8 ORF gene, the embryonic alpha-globin gene π, the distant upstream regulatory element of the α-globin gene domain (MRE), and a region located centrally from the erythroid-specific enhancer were much more abundant within the nuclear matrix DNA than were fragments containing the erythroid-specific enhancer itself, and the globin genes αD and αA. Some other regions studied in the above-mentioned experiments were also underrepresented within the nuclear matrix DNA. These include the first exon of open reading frame TMEM8, the erythroid-specific regulatory element located in a hypersensitive DNAse I 9 Kb upstream from the π gene, and part of a housekeeping gene CGTHBA. Based on the above findings, it is possible to conclude that the area of the genome with coordinates 95-165 Kb (Fig. 1) is not entirely and uniformly attached to the nuclear matrix along its length (Fig. 1). Different levels of representation, within the nuclear matrix DNA, of several individual fragments from this area suggest rather that the area is spatially arranged in micro-loops. Due to their small size, these loops could not be identified following hybridization of BAC-probes with nuclear halos. 

**Fig. 2.**
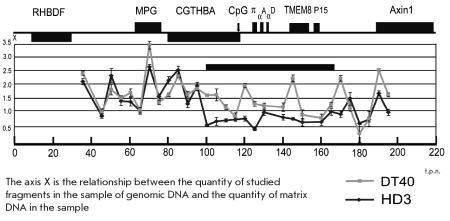
The analysis of relative representation within the nuclear matrix DNA of DNA test-fragments located within genes and various regulatory elements. The positions of test fragments are shown by black horizontal lines under the map of the studied genome area. The sites of DNA cleavage by Bam HI and Bgl I restriction enzymes are shown by long and short vertical lines, respectively, above the scale. Columns in the diagram at the bottom of the figure indicate the relative representation of test fragments within the nuclear matrix DNA.

**Table2:** Primers and TaqMan probes for mapping sites of DNA attachment to the nuclear matrix using real-time PCR

name	sequence
TMEM8 2-8 exon probe	5' FAM CACTGTAACT(TBHQ1)TTGTGTTTTGTGCCTGTAGC 3'
TMEM8 2-8 exon dir	5' AGGCTCCAGCAGTGAGATCC 3'
TMEM8 2-8 exon rev	5' GACCTGGGCATACAAGATAAGC 3'
TMEM 1- exon probe	5' FAM CTACAACAGCCTCACT(BHQ1)GTGAAGCTCTCTC 3'
TMEM 1- exon dir	5' AGGAGCTATCAAATGCAGTGTCT 3'
TMEM 1 exon rev	5' AGGTACAGAAAGGTCCAGAAACA 3'
DHS -9 probe	5' FAM ATTTGATCCTAGATT(BHQ1)GCCAGTGAATTGAA 3'
DHS -9 dir	5' GCGATATTGAATGTTCTCTAGGA 3'
DHS -9 rev	5' GCTTTGTACTGGATGACTGCC 3'
MRE probe	5' FAM AAGTGTTGACT(BHQ1)CATGGTTTGCTAGTTTGC 3'
MRE dir	5' GCTGCCTCATGTTTGTTAAGATA 3'
MRE rev	5' GTGACTCAGCAAGAACAGCAGA 3'
CGTHBA probe	5' FAM TGAACACAGCAGAACT(BHQ1)GGAAGGCAA 3'
CGTHBA dir	5' CACCAGCATGACTAGGTCTTTG 3'
CGTHBA rev	5' ATCAGGACACATGGTTGGACA 3'
CpG probe	5' FAM CCACAAAT(BHQ1)CAAAGCGATGCGGTAT 3'
CpG dir	5' TTCACAGCACAAGGGATAACT 3'
CpG rev	5' GATCTGAGCTGCATCACTAAATG 3'
alphaD probe	5' FAM AACGCCGT(BHQ1)GAAGAACGTGGACAAC 3'
alphaD dir	5' TGTTCACCACCTATCCCCA 3'
alphaD rev	5' GTTGCTCAGCTCAGCCATG 3'
alphaA probe	5' FAM AGGTAGGTGT(BHQ1)CCTTCTCTGTCCTCCG 3'
alphaA dir	5' КAGGGCATCTTCACCAAAATC К3'
alphaA rev	5' GTGGAGCACAGTGAGTCAGG 3'
enh probe	5' FAM AAGTGCTGATGGTTCCT(BHQ1)GTTGGAGTGT 3'
enh dir	5' GCAGACAGGCTGGAGAAGAC 3'
enh rev	5' GGTCATAGCCCAAAGAGCAG 3'
enh right probe	5' FAM TTCAGAGAGTAAGTTCCT(BHQ1)ATGCGTTGCCT 3'
enh right dir	5' TTAGGCTGTGCTCCTCCAAC 3'
enh right rev	5' AACAGGTCGATAAACAGATGCT 3'
Pi probe	5' FAM ACGCAT(BHQ1)GATCCGCACTTGAAATACA 3'
Pi dir	5' GCTCACAGCAGTTTGAAGACCT 3'
Pi rev	5' CAAAAAGCCTGGAGGAGAAC 3'

The spatial organization of the chicken α-globin gene domain has been recently studied using the Chromosome Conformation Capture (3С) experimental approach [14, 15]. It has been found that in HD3 cycling cells (in which globin genes are transcribed at a very low rate) a potentiated chromatin hub is assembled which includes MRE, the CpG island upstream from the α-globin gene domain, and the αD gene promoter. Here we showed that two of the elements of this chromatin hub are associated with the nuclear matrix, which can promote their mutual interaction.

This work was supported by a Presidium of the Russian Academy of Sciences (the Program "Molecular and cellular biology") and the RFBR (grants 09-04-00059, 08-04-91970-NNIOM_a, 08-04-0048a).
